# A Stand-Alone Smart Camera System for Online Post-Earthquake Building Safety Assessment

**DOI:** 10.3390/s20123374

**Published:** 2020-06-15

**Authors:** Ting-Yu Hsu, Xiang-Ju Kuo

**Affiliations:** Department of Civil and Construction Engineering, National Taiwan University of Science and Technology, Taipei 10607, Taiwan; M10605320@mail.ntust.edu.tw

**Keywords:** smart camera system, post-earthquake, building safety assessment, rotation, data fusion

## Abstract

Computer vision-based approaches are very useful for dynamic displacement measurement, damage detection, and structural health monitoring. However, for the application using a large number of existing cameras in buildings, the computational cost of videos from dozens of cameras using a centralized computer becomes a huge burden. Moreover, when a manual process is required for processing the videos, prompt safety assessment of tens of thousands of buildings after a catastrophic earthquake striking a megacity becomes very challenging. Therefore, a decentralized and fully automatic computer vision-based approach for prompt building safety assessment and decision-making is desired for practical applications. In this study, a prototype of a novel stand-alone smart camera system for measuring interstory drifts was developed. The proposed system is composed of a single camera, a single-board computer, and two accelerometers with a microcontroller unit. The system is capable of compensating for rotational effects of the camera during earthquake excitations. Furthermore, by fusing the camera-based interstory drifts with the accelerometer-based ones, the interstory drifts can be measured accurately even when residual interstory drifts exist. Algorithms used to compensate for the camera’s rotational effects, algorithms used to track the movement of three targets within three regions of interest, artificial neural networks used to convert the interstory drifts to engineering units, and some necessary signal processing algorithms, including interpolation, cross-correlation, and filtering algorithms, were embedded in the smart camera system. As a result, online processing of the video data and acceleration data using decentralized computational resources is achieved in each individual smart camera system to obtain interstory drifts. Using the maximum interstory drifts measured during an earthquake, the safety of a building can be assessed right after the earthquake excitation. We validated the feasibility of the prototype of the proposed smart camera system through the use of large-scale shaking table tests of a steel building. The results show that the proposed smart camera system had very promising results in terms of assessing the safety of steel building specimens after earthquake excitations.

## 1. Introduction

After a large earthquake, it is very important to perform damage assessments of affected buildings in order to ensure public safety during aftershocks. Fast and sound post-earthquake evaluations can not only prevent people from returning to unsafe buildings, but also reduce the time spent waiting for experts to determine whether buildings are safe or not, thereby accelerating the recovery of building functions and improving overall earthquake resilience. In practice, the acceleration responses of buildings are often used to calculate the maximum interstory drift during earthquake excitations for post-earthquake damage assessments. For example, Naeim et al. [1] proposed estimating the damage to buildings based on the maximum interstory drifts measured by the accelerometers provided by the California Strong Motion Instrumentation Program and the thresholds provided by the Federal Emergency Management Agency [2].

However, as has been pointed out by many researchers, the low-frequency range of displacements estimated using acceleration signals will be inaccurate when the displacements are not close to a zero mean process because a high-pass filter is required to remove the low-frequency drift after the double integration of acceleration measurements is conducted [3,4]. Trapani et al. [5] proposed solving this problem by simply adding the residual displacement at the end of an excitation to the maximum value of displacement measured by the accelerometers. However, while this results in great improvements in the accuracy of maximum displacement measurements, a maximum error rate of approximately 30% still exists according to numerical simulations of nonlinear single degree of freedom models. This is mainly because the contribution of the nonlinear low-frequency content is still not well considered by simply adding the residual displacement.

Thanks to the rapid advances in computer vision in recent years and the advent of inexpensive high-resolution cameras, the camera-based noncontact approach has emerged as a promising alternative to the use of conventional accelerometers for structural dynamic response measurements. The merits of computer vision-based dynamic displacement measurement include its low cost, ease of setup and operation, and the ability to measure displacements of many locations using a single video measurement. Many studies have already employed computer vision-based approaches for dynamic displacement measurement, damage detection, and structural health monitoring. Most of the techniques in question measure dynamic displacement using cameras located outside the target structures. Among them, the majority of the applications have been in full-scale bridge structures [6,7,8]. For instance, the displacement of a railroad bridge during train crossings was measured by a camera system [9]. As for the application in buildings, only laboratory experiments were conducted. Yoon et al. [10] implemented the Kanade–Lucas–Tomasi tracker for vision-based structural system identification of a laboratory-scale six-story building model using consumer-grade cameras. D. Feng and M.Q. Feng [11] achieved simultaneous measurement of structural displacements at multiple points of a laboratory-scale three-story building model using one camera based on template matching techniques. Chen et al. [12] applied the motion magnification approach proposed by Wadhwa et al. [13] to the modal identification of a simplified building model with high-speed video. As for measurements using in-building surveillance cameras, Hosseinzadeh and Harvey [14] performed an experimental study of a three-story building model to identify modal frequencies using surveillance cameras. Cheng and Kawaguchi [15] tried to measure the peak-to-peak amplitude and frequency of vibration of several high-rise steel buildings during the Great East Japan Earthquake using videos recorded by surveillance cameras. More state-of-the-art literature can be found in a number of review papers [16,17,18,19,20]. However, as pointed out in the review paper by Spencer et al. [20], a number of technical hurdles must be crossed before the use of computer-vision-based techniques for automated structural health monitoring can be fully realized, despite significant progress of computer-vision-based research has been made in recent years. One of the key difficulties is automatically providing more actionable information that can aid in higher-level decision-making.

Meanwhile, there have only been a few studies involving the application of computer vision-based approaches to measure interstory drift using cameras installed inside structures. For instance, Hsu et al. [21] demonstrated the possibility of performing post-earthquake safety evaluations using consumer-grade surveillance cameras installed inside a building. In their study, two cameras in each story were used to measure the maximum interstory drift during earthquakes. According to the results of shaking table tests of a steel building, camera-based measurements are more accurate than accelerometer-based measurements when nonlinear low-frequency displacement occurs during large earthquake excitations, although accelerometer-based measurements are more accurate when a building remains linear during small earthquake excitations. However, the application of camera-based measurements was only validated under the assumption that the cameras are not rotating during the earthquake excitations. This is only possible when the support structures of the cameras are rigid and the floors on which the cameras are mounted are also very rigid or are designed with a special mechanism, such as the hanging floor with pinned connections proposed by Petrone et al. [22]. In contrast, as pointed out by Harvey and Elisha [23], large errors in interstory measurements will be induced by the rotation of cameras installed within a building when the camera support structures are not rigid enough. Therefore, in order to reduce the error in interstory drift measurements resulting from the rotation of cameras during earthquake excitations, the pseudo displacement points (PDP) approach was proposed by Hsu and Kuo [24]. According to the results of shaking table tests of a reinforced concrete building, the errors due to rotational effects of the cameras were substantially decreased when the PDP method was applied. In addition, Lee et al. [25] proposed a vision-based system for the real-time displacement measurement of high-rise buildings using a partitioning approach. It was realized by successive estimation of relative displacements and rotational angles at several floors using a multiple vision-based displacement measurement system. However, for the application using a large number of existing cameras in real buildings, the computational cost of videos from dozens of cameras using a centralized computer becomes a huge burden. Moreover, when a manual process is required for processing the videos, prompt safety assessment of tens of thousands of buildings after a catastrophic earthquake striking a megacity becomes very challenging. Therefore, a decentralized and automatic computer vision-based approach for prompt building safety assessment and decision-making is desired for practical applications.

In this study, we combined a single camera, a single-board computer (SBC), and two accelerometers with a microcontroller unit (MCU) to produce a novel stand-alone smart camera system for fully automatic decentralized video processing and decision-making supporting right after an earthquake event. The PDP method, data fusion algorithm, and necessary artificial neural networks (ANNs) and signal processing algorithms are embedded into the smart camera. Hence, the smart camera has the ability to convert the targets within three regions of interest (ROI) in a video into camera-based displacement time histories of the three targets in real-time. The camera-based interstory drifts without rotational effects of the camera are then obtained using these three time histories and the PDP method. After the vibration has stopped, the final interstory drifts are then calculated using data fusion of the camera-based interstory drifts with the accelerometer-based ones. The level of damage to the story can then be estimated using the maximum interstory drift based on the threshold suggested by the FEMA [2]. The details of the hardware and methodology of the proposed system are explained in Section 2 and Section 3, respectively. An experimental study conducted to validate the smart camera system using large-scale shaking table tests of a steel building is then illustrated in Section 4. Finally, some conclusions are provided in the last section.

## 2. Hardware Design of the Smart Camera System

**Figure 1:**
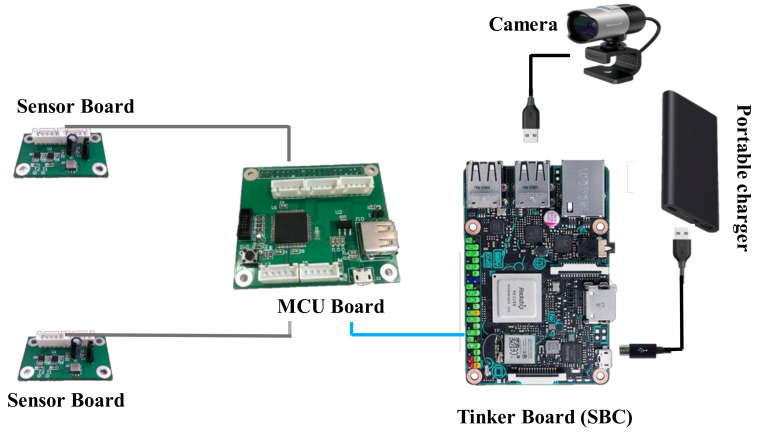
The prototype of the smart camera system.

**Figure 2:**
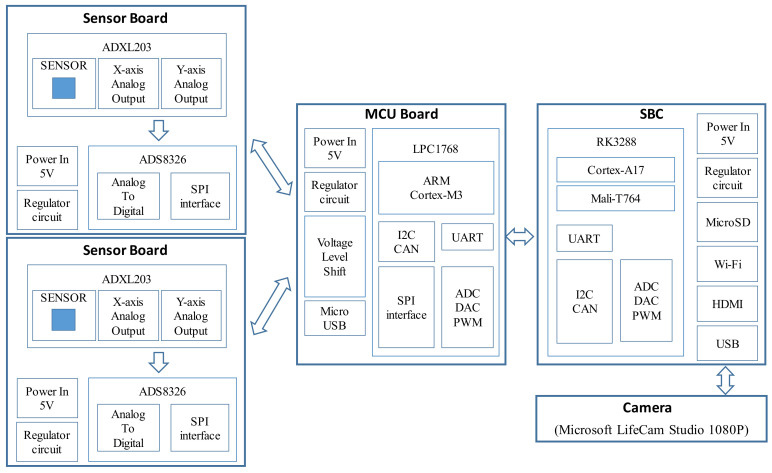
Component diagram of the smart camera system.

The hardware of the smart camera system consists of an SBC, an MCU, two sensor boards with one accelerometer on each board, and a camera. The prototype and main component diagram of the smart camera system are illustrated in Figure 1 and Figure 2, respectively. The video signals of the camera are transmitted to the SBC via the USB interface in real-time. The acceleration signals of the two accelerometers are transmitted to the MCU board first, and then transmitted to the SBC via the UART interface in real-time. The total cost of the smart camera system is approximately USD 400.

The SBC used in this study was the Tinker Board development board based on the ARM architecture. It employs the Quad-core RockChip RK3288 SoC (Rockchip, Fuzhou, China) with ARM Mali^TM^-T764 GPU (ARM Holdings, Cambridge, UK), four USB 2.0, 10/100/1000 Ethernet, WiFi 802.11 b/g/n, Bluetooth 4.0 interface, and 4K H.264 video playback capabilities. The overall performance of the Tinker Board is approximately twice as fast as the Raspberry Pi 3; hence, it was deemed suitable for this study because of the desired real-time processing of video images. In addition, the TinkerOS includes a number of popular applications, e.g., the libraries of Python functions; hence, it offers high versatility and allows for easy programming and development.

The main tasks of the SBC include (a) calculating the short-term average over long-term average (STA/LTA) values of the accelerations to determine the trigger and de-trigger of an earthquake event; (b) converting the movements of targets within three ROIs of the video signals into the dynamic pixel displacements of the three targets based on the patch-based tracking approach, after which the dynamic pixel displacements are converted to the ones in engineering units using the embedded ANNs; (c) calculating the camera-based interstory drift time history without rotational effects of the camera from the three dynamic engineering displacements based on the PDP method; (d) calculating the accelerometer-based interstory drifts by subtracting the accelerations recorded by the floor-mounted sensor board from those recorded by the ceiling-mounted sensor board after double integration and high-pass filtering of the accelerations; (e) performing data fusion of the camera-based interstory drifts with the accelerometer-based ones after time synchronization based on the cross-correlation approach; (f) calculating the fused maximum interstory drift value and then estimating the damage status based on the predefined thresholds. The first four tasks are processed in real-time, while the last two tasks are processed right after the earthquake event is de-triggered. The details of the algorithms used to perform these tasks are described in Section 3.

The camera used in this study was a consumer-grade camera, the Microsoft LifeCam Studio (Microsoft, King County, WA, USA). Its spatial resolution is 1080 p (1920 × 1080 pixel) with an autofocus, wide-angle high-precision glass element lens and CMOS image sensors. The video signals of the camera are transmitted to the SBC via the USB interface in real-time.

The accelerometers used were EVAL-ADXL203 accelerometers (Analog Devices, Norwood, MA, USA). They are two-axis, low-noise, temperature-stable accelerometers with a small size (1” × 1”). The analog signals of the accelerations are converted to digital ones on each sensor board using the 16-bit analog to digital converter ADS8326. The ADS8326 requires very little power, even when operating at its full data rate. Because the ADS8326 transmits the digital signals via the SPI interface, whose communication code is not compatible with the one of the Tinker Board, the MCU board is designed as a relay to transmit the acceleration signals in real-time to the SBC. The MCU board mainly consists of an NXP LPC1768 development board with a 32-bit ARM Cortex-M3, 512 kB flash, 64 kB SRAM, 4 UARTs, USB Device, ultra-low power Real-Time Clock with separate battery supply, and up to 70 general purpose I/O pins. Hence, the real-time operating system can easily transmit the acceleration signals to the SBC in real-time.

## 3. Algorithms of the Embedded Program

The algorithms embedded in the SBC of the smart camera are introduced in this section.

### 3.1. Target Tracking

**Figure 3:**
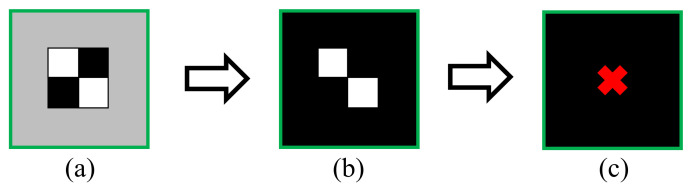
Schematic diagram of (**a**) the region of interest (ROI) containing a cross patch; (**b**) the binary image of the ROI after thresholding; (**c**) the location of the target determined as the centroid of white areas.

On-board real-time processing of the raw video is required to be conducted in the smart camera system within each frame interval because no video will be recorded in the stand-alone system during an earthquake event. Since the computational resources in the SBC board are limited, the dynamic pixel displacements of the target points within the ROIs in the raw video are tracked based on a patch-based approach that requires limited computational efforts, but not other non-target-based methods which require much more computational resources. The targets of the video are the cross patches attached on the floor and on the ceiling, as shown in Figure 3a. The ROIs covering the targets are selected manually. Each of the frames in the raw video is converted to grayscale, and then a binary image of the frame can be obtained by thresholding, as shown in Figure 3b. The threshold that minimizes the intra-class variance of the black and white pixels is determined by the Otsu approach [26]. The pixel locations of the target are determined as the centroids of white areas within the ROIs in each frame, as shown in Figure 3c. In order to achieve better target tracking accuracy and keep the real-time processing smooth at the same time, a 4 × 4 subpixel resolution is applied to the three ROIs in each frame.

**Figure 4:**
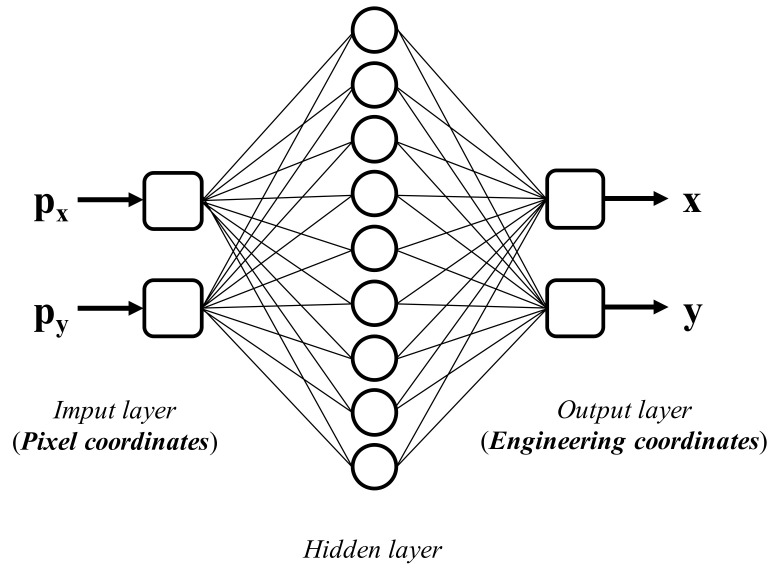
The structure of the artificial neural network (ANN) used to convert the pixel coordinates to the engineering coordinates.

Before the cross patches are attached on the floor and ceiling, a chessboard panel is placed within the ROI for calibration. The displacement between the camera and the target points is basically only two-dimensional; in other words, it is located within the plane of the chessboard. The size of the chessboard, which consists of 9 × 9 intersections with the sides of each block being 1.85 cm in length, covers the area of possible displacement during an earthquake excitation. The mapping between the pixel coordinates $\left(p_{x},p_{y}\right)$ and engineering units (x,y) is constructed using an ANN, as illustrated in Figure 4. The general backpropagation algorithm of feedforward neural networks whose structure is 2 × 9 × 2 with sigmoid activation functions is trained using the 81 pairs of pixel coordinates and the 81 pairs of corresponding engineering units as the input data and output data, respectively. In order to achieve better accuracy of calibration, a 4 × 4 subpixel resolution is applied. The 81 pairs of pixel coordinates are determined using the convolution calculation of a 21 × 21 convolution kernel after the 4 × 4 subpixel resolution is applied.

### 3.2. Rotational Effect Compensation

The pseudo displacement points (PDP) approach was proposed by Hsu and Kuo [24] as a method for reducing the error of interstory drift measurements caused by the rotation of cameras during earthquake excitations. In this study, we embedded the algorithms of the PDP method in the SBC to calculate the interstory drifts measured by the camera in real-time. The procedures of the PDP method are summarized in this subsection.

Specifically, the displacement calculated using the camera images $\Delta f_{i}^{c}(\Delta x_{f_{i}}^{c},\Delta y_{f_{i}}^{c})$ includes (a) the true relative displacement between the target point and the camera $\Delta f_{i}^{t}(\Delta x_{f_{i}}^{t},\Delta y_{f_{i}}^{t})$ and (b) the pseudo relative displacement due to the camera’s rotation $△f_{i}^{r}(△x_{f_{i}}^{r},△y_{f_{i}}^{r})$. In the PDP method, in addition to the target point on the floor, two reference points connected to the ceiling where the camera is mounted are required. It is assumed that the relative displacement of these two target points during an earthquake is solely caused by the camera’s rotation; hence, the camera’s rotational effects can be compensated for using the pseudo displacement of these two target points on the ceiling due to the rotation of the camera.

The first step of the PDP method consists of calculating the initial position of the virtual point using the cross product of the known positions of the two target points on the ceiling, as in Equation (1):

(1) $$c_{0}=a_{0}\times b_{0}$$

where $a_{0}(x_{a_{0}},y_{a_{0}},z_{a_{0}})$ and $b_{0}(x_{b_{0}},y_{b_{0}},z_{b_{0}})$ are the known initial positions of the two target points on the ceiling.

**Figure 5:**
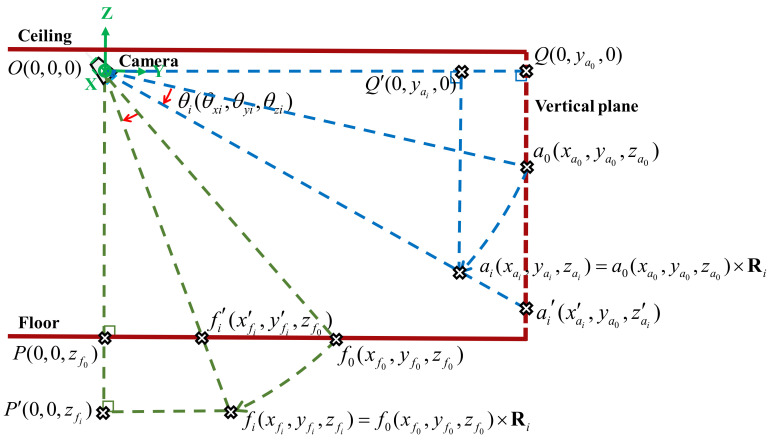
Schematic diagram (side view) describing the geometrical relationships between the camera and the target points whenq the camera rotates.

At time step i, the position of the target point $a_{i}(x_{a_{i}},y_{a_{i}},z_{a_{i}})$ is calculated using Equations (2) and (3), which are derived based on the geometrical relationships shown in Figure 5:

(2) $$\left(△x_{a_{i}},△z_{a_{i}})=({x^{\prime }}_{a_{i}},{z^{\prime }}_{a_{i}})-(x_{a_{0}},z_{a_{0}})=\frac{y_{a_{0}}}{y_{a_{i}}}(x_{a_{i}},z_{a_{i}})-(x_{a_{0}},z_{a_{0}}\right)$$

(3) $$x_{a_{i}}{}^{2}+y_{a_{i}}{}^{2}+z_{a_{i}}{}^{2}=x_{a_{0}}{}^{2}+y_{a_{0}}{}^{2}+z_{a}{{}_{{}_{0}}}^{2}$$

where $\left(△x_{a_{i}},△z_{a_{i}}\right)$ is the displacement of the target point $a_{i}$ measured from the video, and $b_{i}(x_{b_{i}},y_{b_{i}},z_{b_{i}})$ can be obtained in a similar way. Then, the position of the virtual point at time step i is calculated using the cross product again to obtain $c_{i}=a_{i}\times b_{i}$. The rotation matrix at time step i can be calculated using Equation (4):

(4) $${\left[R_{i}\right]}_{3\times 3}=\left[\begin{array}{ccc} x_{a_{i}} & x_{b_{i}} & x_{c_{i}}\\ y_{a_{i}} & y_{b_{i}} & y_{c_{i}}\\ z_{a_{i}} & z_{b_{i}} & z_{c_{i}} \end{array}\right]{\left[\begin{array}{ccc} x_{a_{0}} & x_{b_{0}} & x_{c_{0}}\\ y_{a_{0}} & y_{b_{0}} & y_{c_{0}}\\ z_{a_{0}} & y_{b_{0}} & z_{c_{0}} \end{array}\right]}^{-1}$$

Once the rotation matrix $R_{i}$ is obtained, the position of the target point $f_{i}(x_{f_{i}},y_{f_{i}},z_{f_{i}})$ on the floor at time step i can be calculated using Equation (5):

(5) $${\left[\begin{array}{c} x_{f_{i}}\\ y_{f_{i}}\\ z_{f_{i}} \end{array}\right]}_{3\times 1}={\left[R_{i}\right]}_{3\times 3}{\left[\begin{array}{c} x_{f_{0}}\\ y_{f_{0}}\\ z_{f_{0}} \end{array}\right]}_{3\times 1}$$

where $f_{0}(x_{f_{0}},y_{f_{0}},z_{f_{0}})$ is the known initial position of the target point on the floor. Then, the pseudo displacement of the target point $△f_{i}^{r}=(△x_{f_{i}},△y_{f_{i}})$ at time step i due to the rotational effect can be obtained using Equations (6) and (7):

(6) $$△f_{i}^{r}(△x_{f_{i}}^{r},△y_{f_{i}}^{r})=({x^{\prime }}_{f_{i}},{y^{\prime }}_{f_{i}})-(x_{f_{0}},y_{f_{0}})=\frac{z_{f_{0}}}{z_{f_{i}}}(x_{f_{i}},y_{f_{i}})-(x_{f_{0}},y_{f_{0}})$$

(7) $$x_{f_{i}}{}^{2}+y_{f_{i}}{}^{2}+z_{f_{i}}{}^{2}=x_{f_{0}}{}^{2}+y_{f_{0}}{}^{2}+z_{f}{{}_{{}_{0}}}^{2}$$

Finally, the true relative displacement between the target point on the floor and the camera on the ceiling $\Delta f_{i}^{t}(\Delta x_{f_{i}}^{t},\Delta y_{f_{i}}^{t})$ can be obtained using Equation (8):

(8) $$\Delta f_{i}^{t}(\Delta x_{f_{i}}^{t},\Delta y_{f_{i}}^{t})=\Delta f_{i}^{c}(\Delta x_{f_{i}}^{c},\Delta y_{f_{i}}^{c})-△f_{i}^{r}(△x_{f_{i}}^{r},△y_{f_{i}}^{r})$$

The interstory drift time history, i.e., the true relative displacement between the target point and the camera at each time step during the earthquake excitation, is recorded in the smart camera system for further calculation.

Note that the PDP method assumes there is little relative displacement between the camera and the ceiling where the camera is mounted on. This can be realized in practice provided the support of the camera is rigid and the length of the support is short. As a result, even when there is a small rotation of the ceiling on which the camera is mounted because both the rotation of the ceiling and the length of the support are very small, the translation of the camera due to the rotation of the ceiling will be relatively very small compared to the interstory drift. For instance, when a 1% drift ratio occurred in the story 3000 mm in height, the interstory drift is approximately 30 mm. Assuming the length of the support is approximately 100 mm, the rotation of the ceiling is approximately 1*π*/180, and the translation of the camera due to the rotation of the ceiling will be approximately only 1.75 mm, which is only 5.8% of the interstory drift ratio. Based on this condition, the relative displacement between the camera and the floor is mainly contributed by the interstory drift after the rotation of the camera is corrected. As a result, the error due to rotational effects will be greatly reduced after the PDP method is applied.

### 3.3. Data Fusion

The computational resources in the smart camera are limited; hence, the frame rate of the interstory drifts measured by the camera is approximately 13 Hz. Generally, because the image will be blurred more seriously when the speed of the interstory drift is faster, the error of the interstory drift measurement will be larger. When the frame rate of the video is low, the error of the interstory drift measurement will be even larger, especially when the vibration speed is high during an earthquake with large intensity. Therefore, the accuracy of the interstory drift measurements made by the camera may not be high enough.

On the other hand, because no reference is required when using acceleration to estimate displacement time history in an indirect way, acceleration-based approaches are among the engineering practices used to estimate the interstory drifts of buildings during earthquake excitations (e.g., Naeim et al. [1]). However, when using acceleration to estimate displacement, high-pass filtering is always applied to remove low-frequency drift after the numerical integration of accelerations; hence, the critical nonlinear low-frequency behavior and permanent drift of the buildings are also removed. Considering the advantages and disadvantages of the vision-based and acceleration-based approaches discussed above, some studies have combined low-frame-rate vision-based measurements with high-sampling-rate acceleration-based measurements to achieve accurate displacement measurements [4,27]. In this study, such a fusion approach was employed to obtain the fused interstory drifts.

**Figure 6:**
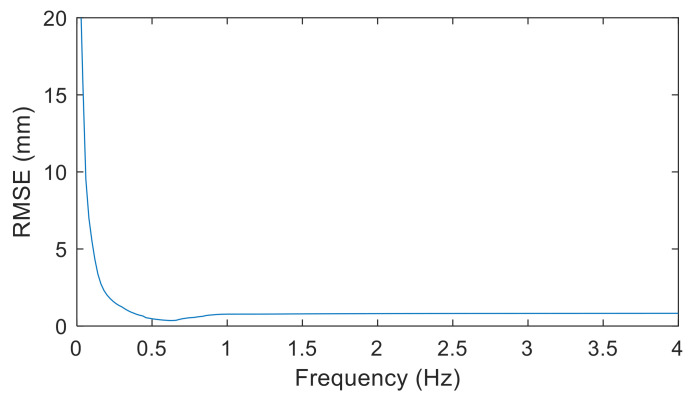
The root mean square error (RMSE) values of the fused interstory drifts using different cut frequencies.

**Figure 7:**
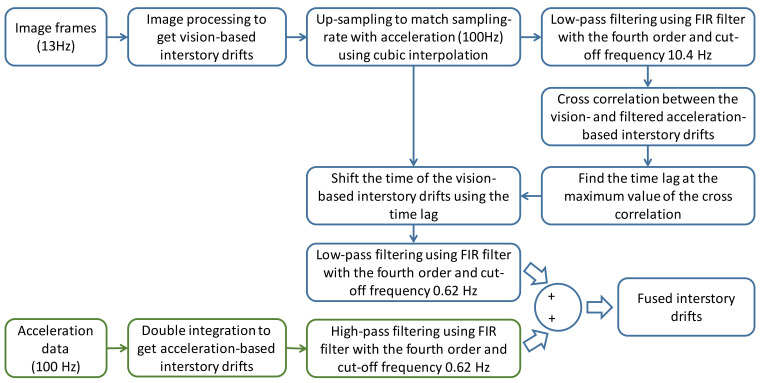
The schematic diagram of the process of data fusion.

The data fusion procedure for combining the vision-based interstory drifts with the acceleration-based interstory drifts is summarized here. The sampling rate of the acceleration data in this study was 100 Hz. Hence, the interstory drifts measured by the camera were upsampled to 100 Hz using cubic interpolation. Cross-correlations of the vision-based and acceleration-based interstory drifts were then calculated, and the time shift with the largest cross-correlation value was used to synchronize the data. An FIR filter with stable poles and a linear phase delay was employed to perform low-pass and high-pass filtering of the vision-based and acceleration-based interstory drifts, respectively. The filter order was designated as four for the data fusion, as suggested by Park et al. [4]. Because nonlinear behavior of a building specimen may occur during earthquake excitations (see Section 3), the cut-off frequency of the filters was determined based on the real behavior of the building specimen during experimental tests. Using the interstory drifts measured by linear variable differential transformers (LVDTs) as reference values, the root mean square error (RMSE) values of the fused interstory drifts using different cut-off frequencies were calculated, and the typical results are shown in Figure 6. As can be seen in Figure 6, it is evident that the cut-off frequency can be determined at the frequency with the smallest RMSE values. The cut-off frequency was determined to be 0.62 Hz for the building specimen. The detail of the process of data fusion used in this study is illustrated in the schematic diagram (Figure 7). The fused interstory drifts were used to estimate the damage states of the building after earthquake excitations.

Note that the appropriate cut-off frequency could be different case by case. In the present study, we measure both the vision-based and acceleration-based interstory drifts together with a baseline measurement of LVDT during earthquake excitations for tuning. In reality, it is not practical to determine the appropriate cut-off frequency using similar approaches. In order to provide suggestions for the determination of the appropriate cut-off frequency for practical applications to real buildings, a large number of numerical studies based on different measurement qualities and structural characteristics are required in future study.

### 3.4. Damage State Estimation

The maximum absolute fused interstory drift during an earthquake excitation was used to estimate the damage state of the building specimen. Five damage states, i.e., no damage, slight damage, moderate damage, extensive damage, and complete damage, as defined by FEMA, are employed to describe the damage state of a building after an earthquake excitation. Different thresholds of interstory drift ratios of different building types, heights, and seismic design levels can be found in the tables of the technical manual of the earthquake model of the multi-hazard loss estimation methodology (HAZUS-MH) [2].

## 4. Steel Building Shaking Table Tests

### 4.1. Experimental Setup

**Figure 8:**
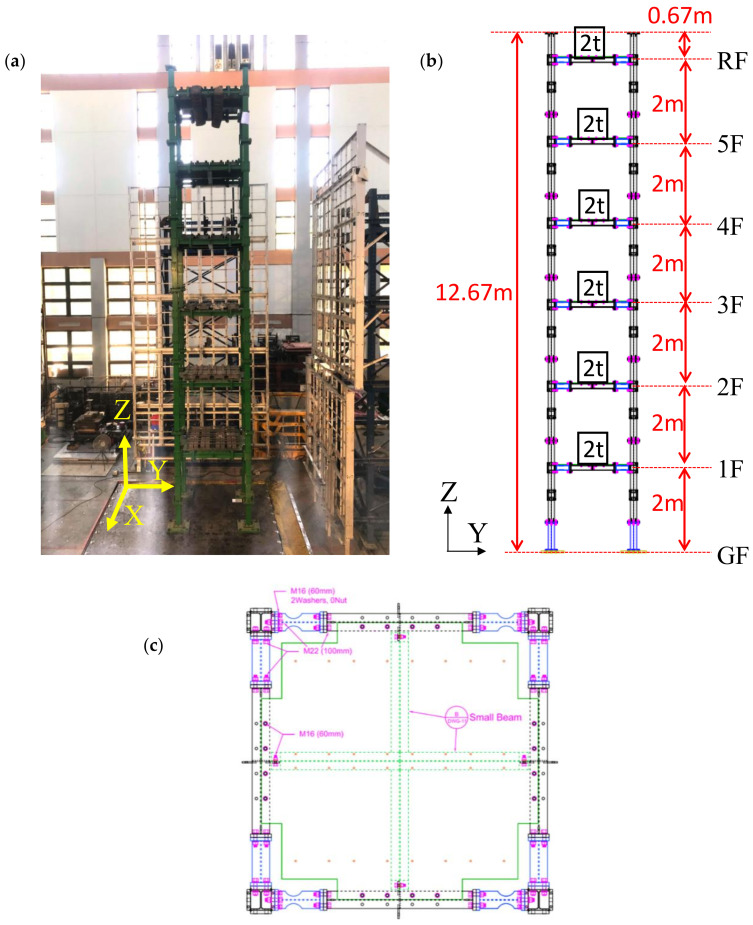
The scaled six-story steel building specimen: (**a**) overview; (**b**) elevation view; (**c**) plan view of first floor with weaker cross section of the beams along the Y-direction.

A scaled six-story steel building structure designed and constructed at the National Center for Research on Earthquake Engineering (NCREE) in Taiwan was used to experimentally test the proposed smart camera system, as shown in Figure 8.

The dimensions of each story of the building were 2.0 m wide × 2.0 m deep × 2.0 m high, and the mass of each floor was 2000 kg. Each beam and column were made of A572Gr50 steel and had a customized I-shaped cross-section with 125 mm × 6.9 mm web and 125 mm × 9 mm flanges. The weak axis of the column was along the X-direction. By installing a weaker cross section of the beams with flanges cut along the Y-direction in the first story (see Figure 8c), the damage location was controlled at the first story. Because the deformation within the upper stories of the building specimen was expected to be very small, the smart camera system was only installed in the first story. The tri-axial 1940 El Centro earthquake was employed to excite the building specimen. The peak ground acceleration (PGA) was scaled from 50 to 250 Gal with an interval of 50 Gal. In total, five shaking table tests were conducted in a sequential manner.

**Figure 9:**
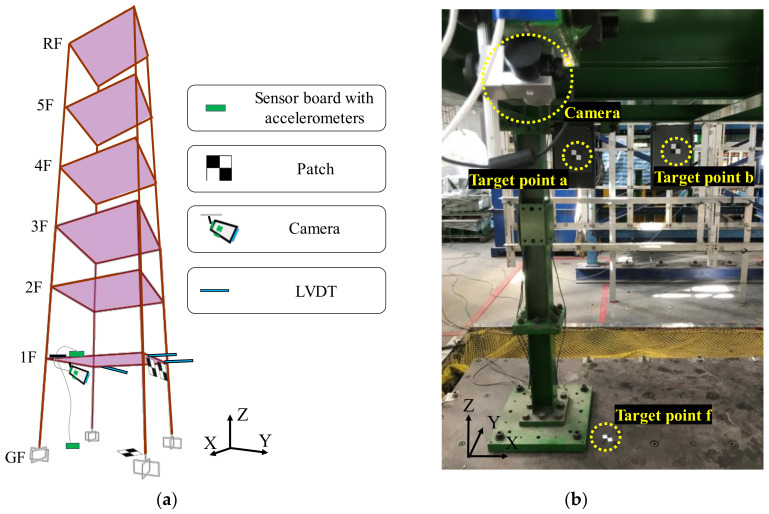
(**a**) Schematic diagram indicating the locations of the target patches, the linear variable differential transformers (LVDTs), and the components of the smart camera system installed in the first story; and (**b**) the target points and the camera.

The camera (Microsoft LifeCam Studio) was mounted on the bottom surface of a beam of the first floor, as shown in Figure 9. The target patch was attached on the ground close to one of the column corners, and two reference patches were attached to vertical plates fixed on the beam of the first floor. The sensor boards with accelerometers were fixed to the corner of the first floor and ground floor close to the camera. The distance between the two reference points on the first floor was approximately 0.5 m, while the distances between the camera and the two reference points were approximately 2.0 m. The distance between the camera and the target point on the ground floor was approximately 2.6 m. In addition to the smart camera system, three LVDTs were placed on the first floor of the building specimen, as shown in Figure 9a. The signals of the LVDTs were digitalized with 200 Hz. We calculated the interstory drifts between the camera and the target points using those measured by these LVDTs based on the rigid floor assumption, and used these drift measurements as references in this study.

According to the HAZUS-MH technical manual, the building specimen belongs to the category of a mid-rise steel moment frame with a high-code seismic design level. Hence, the thresholds of the slight, moderate, extensive, and complete damage levels for the specimen are 0.40%, 0.80%, 2.00%, and 5.33%, respectively.

### 4.2. Results

Due to the limited computational resources in the Tinker Board, recording the video and calculating the interstory drifts during earthquake excitation at the same time would make the frame rate much lower; hence, only the calculated interstory drifts of the target points were recorded.

**Figure 10:**
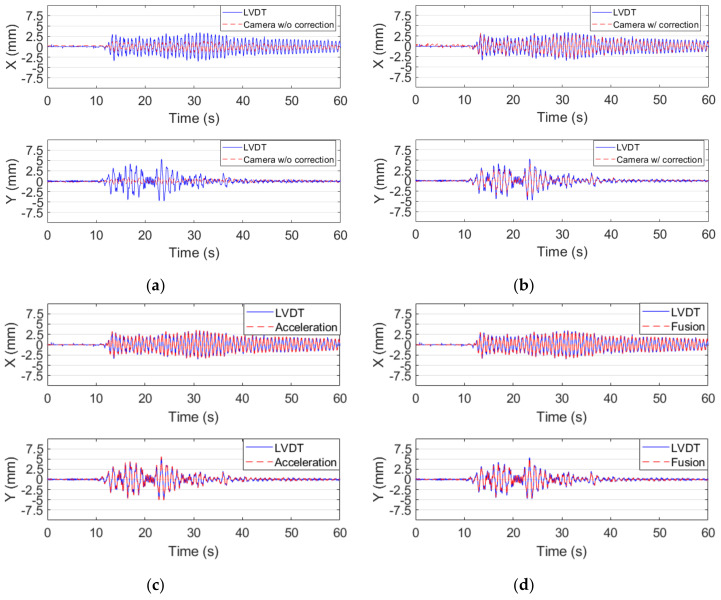
Time histories of interstory drift during the El Centro earthquake excitation with a peak ground acceleration (PGA) of 50 gal measured by: (**a**) the camera without correction; (**b**) the camera with correction; (**c**) acceleration; and (**d**) the camera fused with the accelerations. (upper: X-direction and lower: Y-direction).

**Figure 11:**
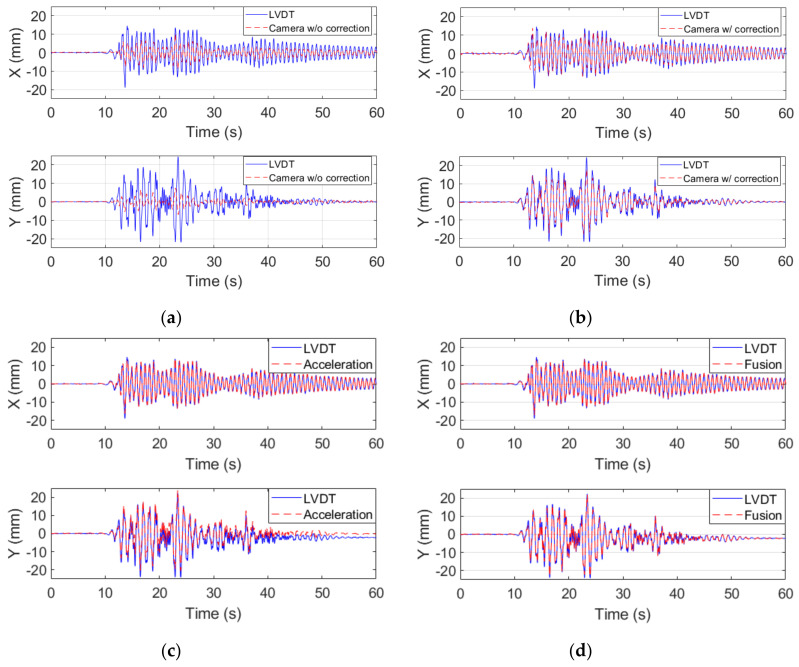
Time histories of interstory drift during the El Centro earthquake excitation with PGA of 250 gal measured by: (**a**) the camera without correction; (**b**) the camera with correction; (**c**) acceleration; and (**d**) the camera fused with the accelerations. (upper: X-direction and lower: Y-direction).

First of all, the calculated interstory drifts between the camera and the target points before the correction of rotational effects were observed. Although five earthquake excitations were conducted, only the interstory drifts of the smallest and the largest excitations, i.e., those with PGA values of 50 gal and 250 gal, respectively, are shown in Figure 10 and Figure 11, respectively, for the sake of conciseness. As can be observed in Figure 10a and Figure 11a, compared to the interstory drifts measured by the LVDTs, the original interstory drifts measured by using the video from the camera without correction of the rotational effects, i.e., $△f_{}^{c}$, were seriously underestimated in both directions. The direction of interstory drifts were even opposite to those measured by the LVDTs in the Y-direction because the interstory drifts in the Y-direction due to the rotational effects were opposite to the actual interstory drifts, and the amplitudes of the interstory drifts due to the rotational effects were even larger than those of the actual ones.

The interstory drifts between the camera and the target points after the correction of the rotational effects using the PDP method, i.e., $△f_{}^{t}$, are shown in Figure 10b and Figure 11b. Evidently, the interstory drifts were much closer to the reference values after the correction of the rotational effects. However, due to the limited frame rate of the smart camera system, the error of the interstory drifts measurements could still be easily observed in the time history diagrams. The acceleration-based interstory drifts during the excitation with PGA equaling 50 gal are plotted in Figure 10c. They look quite close to the ones measured by LVDTs. However, there is evidently a large difference between the interstory drifts in the Y-direction measured by the accelerometers and LVDTs during the excitation with PGA equaling 250 gal, as plotted in Figure 11c. This is because the nonlinear low-frequency content of interstory drifts was removed from the acceleration-based interstory drifts when performing high-pass filter after double integration. Figure 10d and Figure 11d show the fused interstory drifts. After the combination of the acceleration-based and vision-based interstory drifts, the quality of the measured interstory drifts was improved substantially. Even the residual interstory drifts of approximately 2.15 mm were successfully measured by the fused interstory drifts, whereas it was not possible to measure these residual drifts using the acceleration-based approach because the nonlinear low-frequency content of the signals was always removed by the high-pass filter. This illustrated the merit of the fused interstory drifts because the low-frequency content of the signals, including nonlinear low-frequency and residual interstory drifts, could be measured by the vision-based approach.

For post-earthquake damage assessment of the building specimen, the maximum absolute interstory drifts during the earthquake excitations play a critical role. The maximum absolute interstory drifts measured by the smart camera system and the LVDTs are summarized in Table 1, both in mm units and in percentages. The errors of the maximum absolute interstory drifts without correction of the rotational effects during the five earthquake excitations were always underestimated, by between −3.52 and −13.68 mm and approximately −0.18% and −0.68% in drift ratio. After correction of the rotational effects using the PDP method, the errors during the five earthquake excitations were still always underestimated, but to much more reasonable levels of between −0.99 and −5.54 mm and approximately −0.05% and −0.28% in drift ratio. The errors of the maximum absolute interstory drifts measured by the accelerometers during the three smallest earthquake excitations were quite small, between 0.15 and 0.46 mm and approximately 0.01% and 0.02% in drift ratio. However, the ones during the largest two earthquake excitations were quite large, between −1.78 and −2.25 mm and approximately −0.09% and −0.11% in drift ratio, because nonlinear low-frequency content was removed during the high-pass filter process. Finally, the errors of the maximum absolute fused interstory drifts during all the five earthquake excitations were quite acceptable, between −0.85 and 0.38 mm and approximately −0.04% and 0.02% in drift ratio.

Using the maximum absolute fused interstory drifts during the earthquake excitations, the damage level after the excitation of each earthquake was estimated according to the thresholds provided in the HAZUS-MH technical manual. The estimated maximum interstory drift values using the camera without correction, the camera with correction, the accelerometers, and the camera fused with the accelerometers are listed in Table 1. The estimated damage levels using the LVDTs are also listed in Table 2 as references. Evidently, most of the damage levels estimated using the camera without correction were underestimated, and even the moderate damage level was underestimated as safe after the excitation with a PGA value of 150 gal. After correction using the PDP method, most of the damage levels estimated using the camera were identical to the reference ones, except that the damage level after the excitation with a PGA of 150 gal was estimated as light damage, whereas it should have been estimated as moderate damage. Finally, all the damage levels estimated using the fused interstory drifts were identical to the reference ones.

## 5. Conclusions

In this study, the prototype of a novel stand-alone smart camera system for decision-making about building safety after an earthquake was developed. The hardware of the system was designed to enable the online processing of the video to obtain interstory drifts. The PDP algorithm used to compensate for camera’s rotational effects, the algorithm used to track the movement of three targets within three ROIs, the ANNs used to convert the interstory drifts of pixels to engineering units, and some necessary signal processing algorithms, including interpolation, cross-correlation, and filtering algorithms, were embedded in the smart camera system. In addition, both the interstory drifts measured by the video data of the single camera and the acceleration data of two accelerometers were fused together to measure the interstory drifts during an earthquake excitation with high accuracy, even when nonlinear low-frequency and residual interstory drifts existed. By utilizing this approach, the smart camera system has the ability to obtain the maximum interstory drifts during an earthquake right after the earthquake excitation using the decentralized computational resources of the smart camera system itself. Based on the thresholds provided in the HAZUS-MH technical manual, the safety of a building can then be assessed immediately after an earthquake.

The developed prototype of the smart camera system was validated in large-scale shaking table tests of a six-story steel building. In this study, we focused only on establishing a proof of concept for the developed smart camera system; hence, only one set of the smart camera system was installed in the first story of the steel building where the interstory drift was anticipated to be the largest. Based on the results, we concluded that the errors due to rotational effects were decreased substantially after correction using the PDP method, and the errors of the interstory drift measurements were reduced even more when the interstory drifts measured by the camera were fused with the interstory drifts measured by the accelerometers. The damage levels estimated using the maximum absolute fused interstory drifts during the earthquake excitations measured by the smart camera system were identical to the ones measured by the LVDTs, whereas some of the damage levels estimated using the camera with and without the correction of rotational effects were underestimated. Nevertheless, the results show that the developed smart camera system had very promising results in terms of assessing the safety of the steel building specimen after earthquake excitations. As pointed out by the review paper by Spencer et al. [20], the realization of the fully automatic decision-making system is one of the challenges in the realization of vision-based automated inspection and monitoring of civil infrastructure. The proposed stand-alone smart system illustrates the possibility to support decision-making automatically without any manual operation within seconds after an earthquake event once the system is installed and set up well. Some limitations of using the proposed smart camera system are summarized herein: (a) printed targets placed at three locations are required; (b) occlusions of the printed targets are not solved yet; (c) the ROIs needed to be manually selected; (d) manual calibration within the ROIs is required; and (e) the cut-off frequency for data fusion needed to be decided. Further studies are still required to improve the robustness of the system due to these limitations and environmental disturbance, e.g., light change during an earthquake event.

## Figures and Tables

**Table 1:** Maximum interstory drift values and drift ratios during the five earthquake excitations.

PGA	LVDT		Camera w/o Correction				Camera w/ Correction				Acceleration				Fusion			
	Maximum		Maximum		Error		Maximum		Error		Maximum		Error		Maximum		Error	
	Drift	Drift Ratio	Drift	Drift Ratio	Drift	Drift Ratio	Drift	Drift Ratio	Drift	Drift Ratio	Drift	Drift Ratio	Drift	Drift Ratio	Drift	Drift Ratio	Drift	Drift Ratio
	(mm)	(%)	(mm)	(%)	(mm)	(%)	(mm)	(%)	(mm)	(%)	(mm)	(%)	(mm)	(%)	(mm)	(%)	(mm)	(%)
50 gal	5.38	0.27	1.86	0.09	−3.52	−0.18	4.39	0.22	−0.99	−0.05	5.61	0.28	0.23	0.01	5.19	0.26	−0.19	−0.01
100 gal	11.35	0.57	3.89	0.19	−7.46	−0.37	9.55	0.48	−1.80	−0.09	11.81	0.59	0.46	0.02	10.99	0.55	−0.36	−0.02
150 gal	17.37	0.87	7.04	0.35	−10.33	−0.52	13.89	0.69	−3.48	−0.17	17.52	0.88	0.15	0.01	16.85	0.84	−0.52	−0.03
200 gal	23.06	1.15	9.38	0.47	−13.68	−0.68	19.81	0.99	−3.25	−0.16	21.28	1.06	−1.78	−0.09	23.44	1.17	0.38	0.02
250 gal	26.30	1.32	12.65	0.63	-13.65	-0.68	20.76	1.04	-5.54	-0.28	24.05	1.20	-2.25	-0.11	25.45	1.27	-0.85	-0.04

**Table 2:** Estimated damage levels after the five earthquake excitations.

PGA	LVDT	Camera w/o Correction	Camera w/Correction	Acceleration	Fusion	
50 gal	safe	safe	safe	safe	safe	
100 gal	slight	safe	slight	slight	slight	
150 gal	moderate	safe	slight	moderate	moderate	
200 gal	moderate	slight	moderate	moderate	moderate	
250 gal	moderate	slight	moderate	moderate	moderate	
